# Effectiveness of emergency department-based and initiated youth suicide prevention interventions: A systematic review

**DOI:** 10.1371/journal.pone.0289035

**Published:** 2023-12-05

**Authors:** Rebecca Balasa, Sophie Lightfoot, Kristin Cleverley, Robyn Stremler, Peter Szatmari, Zenita Alidina, Daphne Korczak

**Affiliations:** 1 Dalla Lana School of Public Health, University of Toronto, Toronto, Canada; 2 School of Nursing, University of Ottawa, Ottawa, Canada; 3 Lawrence S. Bloomberg Faculty of Nursing, University of Toronto, Toronto, Canada; 4 Temerty Faculty of Medicine, University of Toronto, Toronto, Canada; 5 Centre for Addiction and Mental Health, Toronto, Canada; 6 The Hospital for Sick Children, Toronto, Canada; 7 Temerty Faculty of Medicine, Division of Child and Youth Mental Health, University of Toronto, Toronto, Canada; 8 The Hospital for Sick Children and Cundill Centre for Child and Youth Depression, Centre for Addiction and Mental Health, Toronto, Canada; 9 Department of Psychiatry, University of Toronto, Toronto, Canada; 10 Department of Psychiatry, The Hospital for Sick Children, Toronto, Canada; Universidade Federal do Rio Grande do Sul, BRAZIL

## Abstract

**Objective:**

This systematic review examined the effectiveness of Emergency Department-based and initiated youth suicide prevention interventions for suicide attempts, suicidal ideation, hospitalization, family system functioning, and other mental health symptoms.

**Methods:**

We searched five databases for randomized controlled trial (RCT) studies that examined Emergency Department-based and initiated suicide prevention interventions among youth aged 10 to 18 years old between May 2020 to June 2022. Using Cohen’s d and 95% confidence interval as our standardised metrics, we followed the Preferred Reporting Items for Systematic Review and Meta-Analyses (PRISMA) and Synthesis Without Meta-Analysis in Systematic Reviews (SWiM) guidelines when synthesizing, interpreting, and reporting the findings of this review.

**Results:**

Five studies were included in this review. Findings were first synthesized according to the targeted population of the study intervention and this review’s outcomes. Two interventions were effective for decreasing depressive symptoms, hospitalization recidivism, and/or increasing family empowerment. There were no interventions that reduced subsequent suicide attempts. A meta-analysis was not conducted due to the heterogeneity of the data.

**Conclusion:**

A need exists to develop and evaluate Emergency Department-based and initiated youth suicide prevention interventions that can be successfully and sustainably implemented in practice. Future research should focus on evaluating the components of interventions that effectively mitigate suicide risk among high-risk youth.

## 1. Introduction

Suicide is the leading cause of non-accidental death for youth between 10 to 18 years of age in North America [1, 2]. Marked increases in Emergency Department visits by youth experiencing suicidal ideation or following a suicide attempt [3] have been documented between 2007 to 2015 [4–6] with estimated annual visits having increased significantly in the United States (US) from 58,0000 to 1.12 million [6], without a significant change in total Emergency Department visits during this time [4]. This increase is consistent with earlier National Hospital Ambulatory Medical Care Survey (NHAMCS) analyses which reported a two-fold increase in Emergency Department visits by youth for suicide-related behaviors between 1993 to 2008 [7]. While further data are needed to understand the scope of the COVID-19 pandemic’s impact for suicide-related behaviors and ideation among youth since 2020, emerging evidence suggests that the pandemic response and related psychosocial disruptions may have contributed to increasing mental health concerns for this population [8–10], particularly among gender minoritized youth [11]. Following the first COVID-19 pandemic lockdown in 2020, US Emergency Departments saw an increase in youth presentations for suicide-related concerns, especially among youth who did not have a prior mental health diagnosis [8, 10]. An overall increase of death by suicide was also observed in the first 10 months of the COVID-19 pandemic, disproportionately among young, males, and/or who belong to an ethnic or racial minoritzed group [12]. Data suggest that youth who are discharged from the Emergency Department following a suicide attempt are most at risk to re-attempt suicide within three months and that the risk of mortality on re-attempt is approximately 10-fold higher when compared to that of their peers who did not have a prior attempt [13]. Together, these analyses provide evidence of an alarming acceleration in pediatric presentations to the Emergency Department for suicide-related concerns [4, 7]; thus, requiring further attention to the unique needs of youth presenting to the Emergency Department with these presentations.

Given the clinically significant increase in youth Emergency Department presentations for suicide-related concerns, it is essential that effective prevention interventions be identified, developed, and implemented within this context. Clinicians and researchers have generated a call to action for evidence-informed guidelines to reduce suicide risk and prevent death by suicide [14]; however, few empirically validated interventions across settings have been shown to reduce suicide-related behaviors and suicidal ideation among youth [14–17]. Even fewer studies have specifically addressed the intervention needs of acutely suicidal youth presenting to the Emergency Department, with only one systematic review published in 2010 [18] and one rapid review published in 2022 [19] reporting on this to date. The systematic review conducted by Newton et al. (2010) aimed to evaluate the effectiveness of Emergency Department-initiated interventions among pediatric patients presenting with suicide concerns [18], while the rapid review conducted by Virk et al. (2022) narrowed the scope of their research to evaluate brief interventions delivered in pediatric Emergency Departments for managing suicidal ideation [19]. This current systematic review provides an updated synthesis of current knowledge on Emergency Department-based and initiated youth suicide prevention interventions and their effects on our primary outcome, suicide-related behaviors, as well as our secondary outcomes, suicidal ideation, other mental health symptoms, hospitalization, and family functioning.

## 2. Methods

A systematic review of randomized controlled trial (RCT) studies that examined Emergency Department-based or Emergency Department-initiated youth suicide prevention interventions was conducted. This review was registered in PROSPERO (CRD42020181270) and followed the Preferred Reporting Items for Systematic Review and Meta-Analyses (PRISMA) [20] and Synthesis Without Meta-Analysis in Systematic Reviews (SWiM) [15] guidelines. We used the SWiM guidelines for reporting findings of this systematic review that did not meet criteria for meta-analysis [21].

### 2.1 Search strategy and selection criteria

The primary author (RB) systematically searched for published peer-reviewed studies in MEDLINE, EMBASE, CINAHL, PsycINFO, and CENTRAL. In consultation with a health sciences librarian at the University of Toronto, the search strategy (S1 Appendix) was created to search for published studies with the following inclusion criteria: (1) RCT study design that randomized study participants to receive an Emergency Department-based or initiated suicide prevention intervention, enhanced treatment-as-usual (ETAU), or treatment-as-usual (TAU) (as defined by each individual study); (2) contained a suicide prevention intervention delivered or initiated in an Emergency Department setting (pediatric, adult, and/or general Emergency Department); (3) enrolled participants between 10 to 18 years of age; (4) reported on participants presenting to an Emergency Department for suicide-related behaviors and/or suicidal ideation or who presented to an Emergency Department for non-psychiatric-related reasons and screened positively for suicide-related behaviors and/or suicidal ideation; and (5) was available in French or English. Studies which included participants outside the set age limits, and that met all other eligibility criteria, were included if the mean age of the study participants was within the included age range. Studies that combined recruitment sources such that the Emergency Department was one of several other, non-acute, potential points of recruitment, and presented aggregated study results (i.e., did not present results of participants recruited from the Emergency Department separately from those that were recruited from a different study site) were not included in this review. We did not restrict study searching or eligibility by year of publication. The initial study searching was conducted between February to May, 2020. An updated search was conducted on May 27^th^, 2022.

### 2.2 Selection process

Articles extracted from the searched databases were uploaded into Covidence [22]. Two independent reviewers (RB;SL) screened titles and abstracts of extracted articles, with discrepancies resolved by consensus. Reviewers achieved a 97% inter-rater agreement for the initial search and a 94% inter-rater agreement for the updated search after screening titles and abstracts. Full text review occurred in the same manner. Reviewers achieved a 93% inter-rater agreement for the initial search and a 97% inter-rater agreement for the updated search after full text screening. Decisions made throughout the screening process were tracked and logged. The selection process for the initial search occurred from May to June, 2020, and between May to June, 2022 for the updated search.

### 2.3 Data extraction and analysis

Full text screening and extraction occurred for the initial search from June to July 2020, and in June of 2022 for the updated search. An Excel spreadsheet was used to manage and track information from extracted articles. To synthesize the findings, we first grouped the studies according to the population for which the study’s intervention was intended (i.e., youth-targeted versus family-targeted). We then grouped the studies’ outcomes according to the primary and secondary outcomes of this review. When extracting data, we defined suicide-related behaviors as a person’s intentional actions that may cause death [23]. These included, but were not limited to, a suicide attempt (i.e., self-injurious behaviors in the presence of any level of intent to die) and deliberate self-harm (i.e., self-injurious behaviors such as a suicide attempt and non-suicidal self-injury) [23]. We defined suicidal ideation as either passive (i.e., thoughts about death or of not being able to carry on) during which a person does not consider ending their own life, nor intends to attempt suicide, or active (i.e., thoughts about ending one own’s life) during which the person considers acting to end their life [23]. Data extraction for other mental health symptoms was individual to each study and included results relating to depression or depressive symptoms, hopelessness, and substance use. Extracted data relating to hospitalization focused on results pertaining to hospitalization recidivism. Finally, given that the population of interest are pediatric patients, we extracted data on family functioning from studies that measured the effects of the suicide prevention intervention on family systems.

Using an effect size calculator, we calculated the Cohen’s d and 95% confidence interval for study outcomes, where possible, as our standardised metrics [21] to determine differences in intervention outcomes between the intervention and control groups post-intervention and to evaluate the effectiveness of the suicide prevention intervention on the outcome.

### 2.4 Risk-of-bias appraisal

Two independent reviewers (RB;SL) assessed the methodological quality of the selected studies based on the recommendations of the Cochrane Collaboration Handbook [24]. The reviewers assessed the following factors for each article included in this review: (1) presence of bias arising from the randomization process (generation of the allocation sequence, adequate concealment, or baseline group differences suggesting a problem with the randomization process); (2) presence of bias due to a lack of, or problems with, blinding; (3) information regarding missing outcome data; (4) concerns regarding the assessment and measurement of outcomes; and (5) reporting of results in accordance with initial analytical plan. The Cochrane Risk of Bias Comparison tool was used in Covidence to assign a level of risk of bias judgement (low risk, unclear, or high risk) [24] by both reviewers. The reviewers met to discuss and resolve discrepancies.

## 3. Results

### 3.1 Studies identified

**Fig 1:**
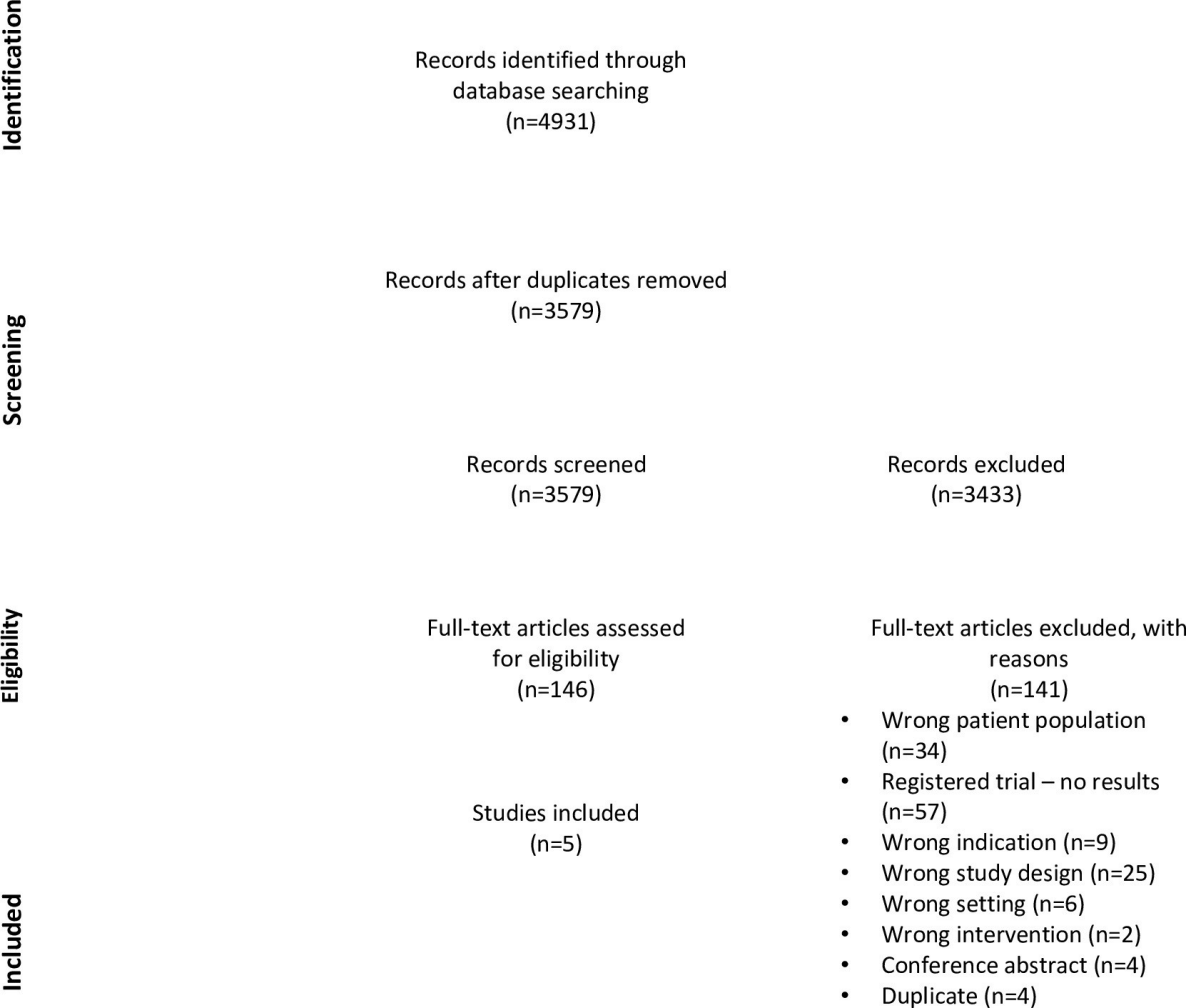
PRISMA flow diagram of studies identified.

The database search yielded 3579 titles and abstracts after duplicates (n = 1352) were removed. This yielded 146 potentially relevant articles for full text screening. Of these, five met inclusion criteria and were included in the final review (Fig 1). Excluded full text articles can be found in S1 Table. The study sample sizes ranged from 49 to 245 (23 to 112 in the intervention group; 26 to 133 in the control group). Two studies trialed an Emergency Department-based or initiated youth suicide prevention intervention among youth participants only [25, 26], while three studies trialed an intervention aimed at youth and their families [15, 27, 28].

All studies were conducted in the United States. Study characteristics are presented in Table 1. A meta-analysis of the extracted data was not possible due to the heterogeneity of the study participants’ clinical presentations and chief complaints, the intervention modalities, the outcomes that were assessed, and the outcome measures. A summary of the interventions’ effect size by outcome can be found in Table 2.

**Table 1:** Study characteristics.

Characteristic	N (%)	Asarnow	Grupp-Phelan	King	King	Wharff
Title of intervention	‐‐	Family Intervention for Suicide Prevention (FISP)	STAT-ED	In-person Follow-up	Teen Options for Change (TOC)	Family Based Crisis Intervention (FBCI)
Sample size at baseline	‐‐	n = 181	n = 159	n = 245	n = 53	n = 142
Date of publication						
2010–2014	2 (40%)	2011		2012		
2015–2019	3 (60%)		2019		2015	2019
Type of publication						
Peer-reviewed journal article	5 (100%)	Peer reviewed	Peer reviewed	Peer reviewed	Peer reviewed	Peer reviewed
Study setting						
Emergency Department	5 (100%)	Emergency Department	Emergency Department	Emergency Department	Emergency Department	Emergency Department
Targeted population						
Youth	2 (40%)			Youth	Youth	
Youth and family	3 (60%)	Youth and family	Youth and family			Youth and family
Emergency Department presentation						
Non-psychiatric related concerns with a positive screen for suicide risk	2 (40%)		Non-psychiatric related concerns with a positive screen for suicide risk		Non-psychiatric related concerns with a positive screen for suicide risk	
Suicide-related concerns	3 (60%)	Suicide-related concerns		Suicide-related concerns		Suicide-related concerns
Study intervention						
Case management	1 (20%)		Case management			
Family empowerment	1 (20%)	Family empowerment				
Crisis therapy	2 (40%)	Crisis therapy				Crisis therapy
Motivational interviewing	2 (40%)		Motivational interviewing		Motivational interviewing	
Provision of resource materials	2 (40%)	Provision of resource materials		Provision of resource materials		
Psychoeducation	2 (40%)			Psycho-education		Psycho-education
Safety planning	2 (40%)	Safety planning				Safety planning
Comparator						
Treatment-As-Usual (TAU)	2 (40%)			TAU		TAU
Enhanced-Treatment-As-Usual (ETAU)	3 (60%)	ETAU	ETAU		ETAU	
Follow-up modality						
In-person	1 (20%)			In-person		
Telephone	4 (80%)	Telephone	Telephone		Telephone	Telephone
Study measures						
Beck Hopelessness Scale	1 (20%)				Beck Hopelessness Scale	
Child Behavior Checklist	1 (20%)	Child Behavior Checklist				
Client Satisfaction Questionnaire	1 (20%)					Client Satisfaction Questionnaire
Conflict Behaviors Questionnaire	1 (20%)	Conflict Behaviors Questionnaire				
FES	1 (20%)					FES
National Institute of Mental Health Diagnostic Interview Schedule for Children Version IV	1 (20%)	National Institute of Mental Health Diagnostic Interview Schedule for Children Version IV				
Recidivism Questionnaire	1 (20%)					Recidivism Questionnaire
RFL-A	1 (20%)					RFL-A
The Aggression Scale	1 (20%)			The Aggression Scale		
Zimmerman’s Delinquency Scale	1 (20%)			Zimmerman’s Delinquency Scale		
AUDIT-C	2 (40%)			AUDIT-C	AUDIT-C	
CES-D	2 (40%)	CES-D	CES-D			
CSSR-S	2 (40%)		CSSR-S		CSSR-S	
RADS-2	2 (40%)			RADS-2	RADS-2	
Service Assessment for Children and Adolescents	2 (40%)	Service Assessment for Children and Adolescents	Service Assessment for Children and Adolescents			
SIQ-Jr	3 (60%)		SIQ-Jr	SIQ-Jr	SIQ-Jr	
Analytic method						
ANOVA	1 (20%)			ANOVA		
Kaplan-Meier	1 (20%)		Kaplan-Meier			
Linear mixed model	1 (20%)					Linear mixed model
Multinomial logistic regression	1 (20%)		Multinomial logistic regression			
Univariate analyses	1 (20%)	Univariate analyses				
Fisher exact test	2 (40%)		Fisher exact test			Fisher exact test
Repeated measures analyses of variance	2 (40%)		Repeated measures analyses of variance		Repeated measures analyses of variance	
Intent-to-treat	3 (60%)	Intent-to-treat	Intent-to-treat		Intent-to-treat	
Logistic regression	4 (80%)	Logistic regression	Logistic regression	Logistic regression		Logistic regression
Any covariates						
No	1 (20%)				No covariates	
Yes	4 (80%)	Covariates	Covariates	Covariates		Covariates
Insurance status	1 (20%)					Insurance status
Public assistance status	1 (20%)			Public assistance status		
Age	2 (40%)	Age	Age			
Gender	2 (40%)	Gender		Gender		
Sex	2 (40%)		Sex			Sex
Race	3 (60%)		Race	Race		Race

Abbreviations: RADS-2, Reynolds Adolescent Depression Scale, 2^nd^ Edition; SIQ-Jr, Suicidal Ideation Questionnaire-Jr; AUDIT-C, Alcohol Use Disorders Identification Test-Concise; C-SSRS, Columbia Suicide Severity Rating Scale; CES-D, Centre for Epidemiological Studies-Depression; RFL- A, Reasons for Living Inventory for Adolescents; FES, Family Empowerment Scale.

**Table 2:** Summary of findings: Effect size by outcome.

Outcome	Baseline	Last Follow-up
Suicidal Ideation		
Grupp-Phelan 2019	d = 0.02, 95% CI: -0.29–0.33	d = 0.18, 95% CI: -0.24–0.60
King 2012	‐‐	d = 0.13, 95% CI: -0.12–0.38
King 2015	d = 0.07, 95% CI: -0.44–0.64	d = -0.16, 95% CI: -0.74–0.42
Wharff 2019	d = -0.10, 95% CI: -0.44–0.23	d = -0.01, 95% CI: -0.34–0.33
Depression		
Asarnow 2011	‐‐	‐‐
Grupp-Phelan 2019	d = -0.03, 95% CI: -0.34–0.29	d = 0.10, 95% CI: -0.32–0.52
King 2012	‐‐	d = 0.21, 95% CI: -0.05–0.46
King 2015	d = -0.28, 95% CI: -0.84–0.29	d = -1.25, 95% CI: -1.89- -0.62
Hopelessness		
King 2015	d = -0.15, 95% CI: -0.73–0.39	d = -0.55, 95% CI: -1.14–0.04
Alcohol Misuse		
King 2012	‐‐	‐‐
King 2015	d = 0.02, 95% CI: -0.55–0.58	d = -0.21, 95% CI: -0.79–0.37
Family System Functioning		
Asarnow 2011	‐‐	d = 0.10, 95% CI: -0.19–0.39
Wharff 2019	d = 0.18, 95% CI: -0.16–0.52	d = 0.53, 95% CI: 0.17–0.88

Cohen’s d = the measured effect of the study’s intervention by outcome

Of the two youth targeted trials, one study examined the effect of communication about the type of follow-up that the youth would receive on their response to a self-reported suicide risk screen (primary outcome) [25]. The authors hypothesized that informing youth of greater in-person staff follow-up prior to them completing the self-report measures would be associated with lower levels of self-reported suicide risk factors [25]. Youth randomized to the intervention group (n = 112) participated in a 20-minute in-person follow-up session with a trained research staff, and youth randomized to the control group (n = 133) received treatment-as-usual (TAU) with no in-person follow-up [25]. The second youth targeted trial examined the effectiveness of the Teen Options for Change (TOC) intervention among youth seeking non-psychiatric care in the Emergency Department, but who screened positively for suicide risk [26]. Youth participants randomized to the intervention group (n = 23) participated in a 35- to 45-minute adapted motivational interview with a mental health professional, while youth in the control group (n = 26) received enhanced treatment-as-usual (ETAU) which was enhanced with a crisis card for suicidal emergencies and written information about depression, suicide risk, firearm safety, and local mental health services [26].

Of the three studies that trialed an Emergency Department-based or initiated youth suicide prevention intervention aimed at youth and their families, the study that evaluated the Family Intervention for Suicide Prevention (FISP) intervention had youth in the intervention group (n = 89) complete a brief youth and family crisis therapy session in the Emergency Department [15]. Family support was strengthened by encouraging youth and parents to identify positive attributes about themselves and each other [15]. Structured telephone contacts for motivating and supporting outpatient treatment attendance were made within the first 48 hours after discharge from the Emergency Department and at one, two, and four weeks post-discharge [15]. Participants randomized to the control group (n = 92) received ETAU which was enhanced by training sessions for Emergency Department staff [15].

A second study examined whether a motivational interviewing–based intervention (STAT-ED) increased linkages to outpatient mental health services and reduced depressive and suicide-related symptoms [27]. Youth were invited to participate if they screened positively for suicide risk while presenting to the Emergency Department for non-psychiatric-related concerns [27]. Participants randomized to the intervention group (n = 80) completed a brief motivational interview targeting mental health care-seeking behaviors and referrals [27]. STAT-ED participants also received case management via telephone call follow-ups to speak about problems for linkages to outpatient mental health treatment [27]. Participants randomized to the control group (n = 79) received ETAU which was enhanced by a brief mental health consultation and referral [27]. Finally, a third study reported efficacy outcomes of a Family Based Crisis Intervention (FBCI) for suicidal youth and their families [28]. Participants randomized to the intervention group (n = 68) completed a 60- to 90-minute session with a clinician in which a joint crisis narrative of the problem was created by the youth and parent [28]. Participants randomized to the control group (n = 71) received TAU [28].

### 3.2 Suicide attempt

Two studies assessed subsequent suicide attempts following a family-based suicide prevention intervention [15, 27]. Neither study’s intervention demonstrated a statistically significant decrease in reported suicide attempts. The STAT-ED study examined whether a motivational interviewing–based intervention increased linkages to outpatient mental health services and reduced depressive and suicide-related symptoms [27]. Following the family-based STAT-ED intervention, three (5.30%) youth in the intervention group (n = 80) and one (2%) youth in the control group (n = 79) reported having attempted suicide (p = 0.62) [27].

The second study evaluated the Family Intervention for Suicide Prevention (FISP) intervention for preventing suicide among youth [15]. Similarly to the STAT-ED intervention, following the FISP intervention, four youth (6%) from the intervention group (n = 89) and five (6%) youth from the control group (n = 92) reported having attempted suicide; one youth had died by suicide, although the group in which this youth was assigned was not reported [15].

### 3.3 Suicidal ideation

Four studies assessed suicidal ideation among youth [25–28]. None of these studies’ interventions resulted in a statistically significant decrease in suicidal ideation. A study that examined the effectiveness of the Teen Options for Change (TOC) intervention among youth seeking non-psychiatric care in the Emergency Department, but who screened positively for suicide risk, identified a non-statistically significant but clinically relevant effect for reducing suicidal ideation following the TOC intervention (d = -0.16, 95% CI: -0.74–0.42) (p = 0.58) [26]. However, this positive effect cannot be definitively attributed to the intervention because there was also a statistically significant effect across groups over time (p<0.01) demonstrating an overall decrease in youths’ suicidal ideation over the study period [26].

A statistically significant decrease in suicidal ideation across groups over time was also observed in two of the other included studies, including the STAT-ED study [27] and the Family Based Crisis Intervention (FBCI) study [28], a family-based intervention that reported suicide-related efficacy outcomes for suicidal youth.

### 3.4 Hospitalization recidivism

Only one study assessed hospitalization recidivism following the FBCI intervention [28]. This study found that patients randomized to the FBCI intervention were significantly more likely to be discharged home from the Emergency Department and reported significantly less hospitalization recidivism [28]. In this study, 26 (38%) youth who received the intervention and 48 (68%) youth in the control group were hospitalized at follow-up (OR: 3.4; 95% CI: 1.7–6.8; p<0.005), indicating a statistically significant association with decreased hospitalization recidivism among youth who received the FCBI intervention [28]. This study used a self-reported questionnaire in which youths’ parents were asked: “Since your initial visit to the ED, has your child required another crisis evaluation?”; and “Since your initial visit to the ED, has your child been psychiatrically hospitalized again?” [28].

### 3.5 Other mental health-related concerns

Four studies reported on the effectiveness of suicide prevention interventions on other mental health-related concerns [15, 25–27], including depressive symptoms, hopelessness, and alcohol misuse.

#### 3.5.1 Depression

Four studies reported on depression or depressive symptoms [15, 25–27], although measurement tools varied, thereby contributing to the heterogeneity of the data. Two studies used the short form Reynolds Adolescent Depression Scale, 2^nd^ Edition (RADS-2) [25, 26], whereas the other two studies used the Centre for Epidemiological Studies-Depression (CES-D) scale [15, 27]. Only the TOC intervention study demonstrated a significant effect for decreasing depressive symptoms from baseline to follow-up among the intervention group (d = 1.07), as well as a significant time*intervention group interaction (p<0.01) [26]. Notably, two family-based interventions reported significant improvements in depressive symptoms across groups and over time [27, 28].

#### 3.5.2 Hopelessness

Only one study assessed hopelessness among youth using the Beck Hopelessness Scale at baseline and following the TOC intervention [26]. The results of this study did not demonstrate a significant intervention or group effect for this outcome (d = 0.40) [26].

#### 3.5.3 Alcohol misuse

Two studies assessed alcohol use, both using the Alcohol Use Disorders Identification Test–Concise (AUDIT-C) measurement tool [25, 26]. Both studies, which included an RCT examining the effect of communication about the type of follow-up that the youth would receive on their response to a self-reported suicide risk screen [25] and the TOC intervention [26], reported non-statistically significant intervention effects for alcohol misuse among youth who received a youth-targeted suicide prevention intervention. Neither study reported a decrease in alcohol misuse across groups over time [25, 26].

### 3.6 Family system functioning

Two studies assessed family system functioning among youth and their families following a family-based intervention [15, 28]. Different measurement scales were used in each study–the Family Empowerment Scale [28] and the Conflict Behaviours Questionnaire [15]–which contributed to the heterogeneity of the data. Following the FBCI intervention, the intervention group demonstrated significantly greater improvements in family empowerment (p<0.01) [28]. Higher scores for family empowerment were also found following the FISP intervention, where family support was strengthened by encouraging youth and parents to identify positive attributes about themselves and each other; however, they were not statistically significant [15].

### 3.7 Risk-of-bias quality appraisal

**Fig 2:**
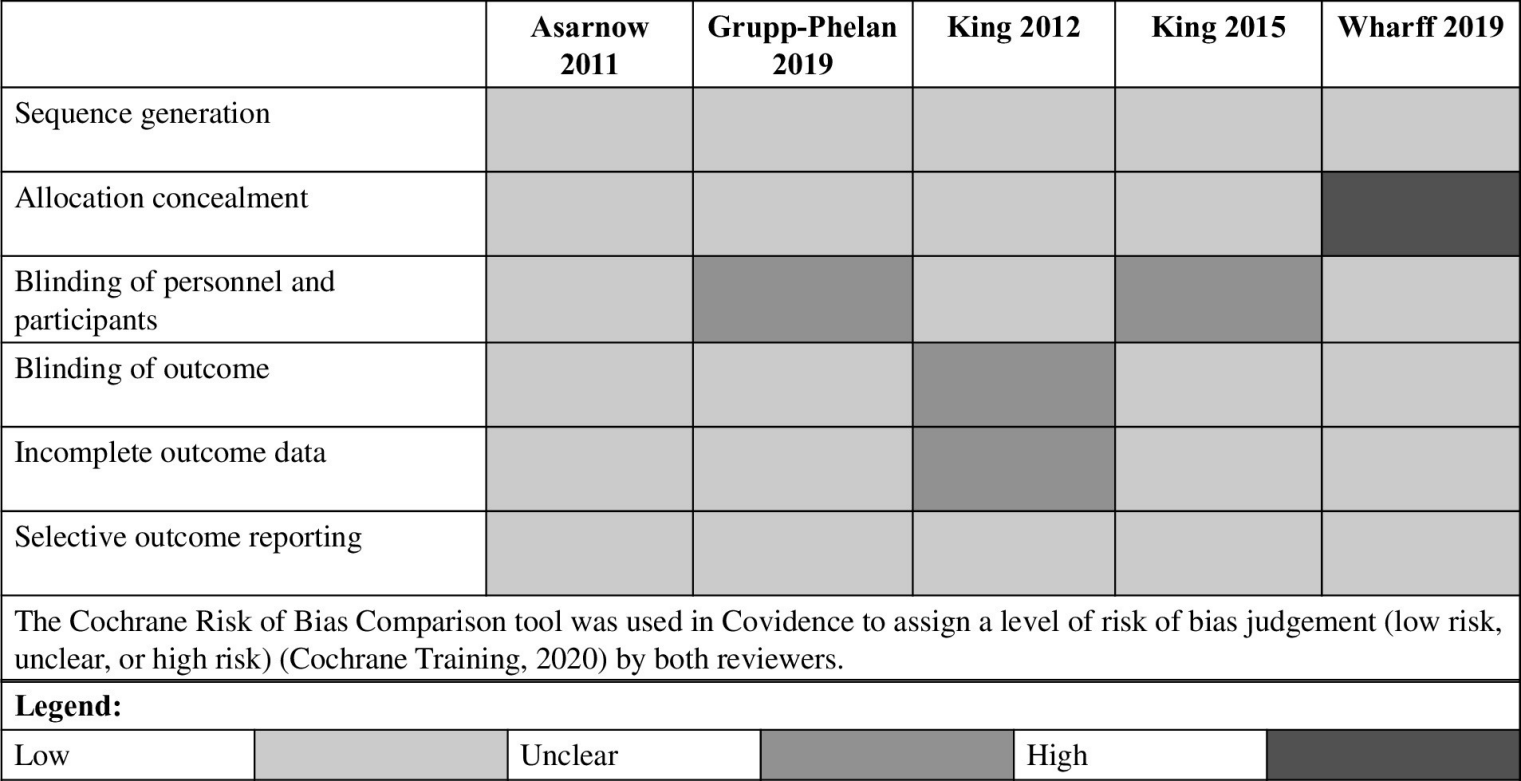
Risk-of-bias quality appraisal of included studies.

As illustrated in Fig 2, four studies had low overall RoB [15, 25–27], although two were missing information regarding the blinding of personnel [26, 27] and one was missing information regarding outcome data [25]. The other study had high RoB identified for allocation concealment [28].

## 4. Discussion

This systematic review presents data synthesizing Emergency Department-based or intitiated youth suicide prevention interventions and their effects on suicide-related behaviors, suicidal ideation, other mental health symptoms, hospitalization, and family functioning. All RCT studies exploring youth suicide prevention interventions implemented or initiated in the Emergency Department are from the United States, demonstrating a need for global health research that investigates youth suicide prevention in the Emergency Department context across health systems and cultures [15, 25–28]. The results of the five studies included in this review suggest that Emergency Department-based or intiatiated youth suicide prevention interventions that have been studied to date have not demonstrated an effect on suicide attempts or other mental health symptoms, with the possible exception of depression [15, 25–28]. Importantly, however, studies have had small sample sizes; only two studies (Teen Options for Change (TOC) and Family Intervention for Suicide Prevention (FISP)) reported statistical significance between the intervention and control group outcomes [15, 27]. The youth-targeted intervention, TOC, found a statistically significant decrease in depressive symptoms following an adapted motivational interview, a co-created action plan, and a telephone follow-up for the youth following discharge from the Emergency Department [26]. Despite the interconnectedness of suicidality with depression, the TOC intervention was not effective for decreasing the frequency of suicidal ideation solely among intervention group participants and did not measure subsequent suicide attempts [26].

Similarly, other studies measuring suicidal ideation found a decrease in frequency across groups and over time [25–28]. It is therefore unknown if the intervention groups’ decrease in suicidal ideation is attributable to the study interventions alone. Furthermore, the TOC intervention was not effective for decreasing alcohol misuse, which is an important indicator for suicide risk [26] when measured in tandem with depressive symptoms. Importantly, the RCT study investigating the effects of communication of staff follow-up on youth’s self-reported suicidal ideation found that youth who are of lower socioeconomic status may be more reluctant to disclose distress or to share information about behavioral problems due to fear, stigma, and social desirability; thus, creating greater barriers for this already vulnerable population to be effectively screened for suicide risk and receive treatment [25].

The Family Based Crisis Intervention (FBCI) was more effective for decreasing subsequent hospitalizations and increasing family empowerment when compared to TAU; however, participants in the intervention group for this study reported greater family empowerment at baseline as well as at follow-up, compared to those in the control group [28]. Further, this study scored high for allocation concealment RoB based on research team members and Emergency Department psychiatrists’ knowledge of the randomization of participants with whom they intervened before and during the study intervention [28]. It is unknown how this may have affected the relational dynamics during the FBCI intervention. Nevertheless, family system functioning has increasingly become a critical component for youth suicide prevention interventions [29–31]. Given that pediatric Emergency Departments commonly practice using the family-centered model of care [32], in which patients and their families are integral members of the healthcare team, family-targeted suicide prevention interventions should be considered for this context.

No interventions were found to have a significant effect on decreasing suicide attempts among youth [15, 27]. Several parents of youth who received the STAT-ED intervention reported difficulty initiating follow-up appointments within the recommended two-month timeframe due to logistic and scheduling barriers, such as long waitlists [27]. Although Emergency Department-based and initiated interventions were found to significantly increase linkages to follow-up care in two of the included studies, the community-based interventions also did not demonstrate a significant effect on suicidality or other mental health-related concerns [15, 27]. This suggests that linking Emergency Department patients to existing outpatient treatment is insufficient for decreasing suicide risk. Instead, further research is urgently needed to develop novel, effective interventions that address this important gap in clinical practice.

Many of the interventions in the included studies were based on and/or applied validated tools to screen for and measure suicide risk (e.g., suicidal ideation, previous suicide attempts, depressive symptoms, alcohol misuse). While these tools have reached a high level of acceptability for clinical practice and research, there is limited research exploring the experiences and perspectives of youth and their families who have engaged with these instruments. A United States-based study that examined youths’ experiences of Emergency Department care following a suicide attempt and the meaning that youth attribute to suicidality found that feelings of ambiguity and “flooding” of thoughts are barriers to answering the types of closed-ended questions commonly used in screening and risk-related instruments [33]. It is, therefore, possible that this phenomenon may have affected how participants responded to both the baseline and follow-up assessments in the included studies. A qualitative evaluation of RCT studies investigating youth suicide prevention interventions could contribute to understanding why these interventions were not more effective despite having robust theoretical foundations.

## 5. Implications and contributions

The only commonality among the interventions that had significant effects for decreasing either depressive symptoms, hospitalization recidivism, and/or increasing family empowerment is telephone follow-up(s) with the youth and/or their family, following discharge from the Emergency Department [26, 28]. The TOC intervention telephone follow-ups aimed to support and facilitate implementation of the youth’s action plan [26], whereas telephone follow-ups for the FBCI were conducted to assess the youth’s adherence to follow-up treatment recommendations [28]. Although the specific aims of each study intervention’s follow-up varied, the overarching premise was to support youth in completing post-Emergency Department discharge treatment plans and recommendations to address their suicide-related risks on an outpatient and ongoing basis. These findings indicate that post-Emergency Department discharge follow-ups may be an important factor for sustained decreases in depressive symptoms and hospitalizations over time, as well as for increasing family empowerment; an important protective factor against youth suicide [26, 28].

Given the variance in the aims of telephone follow-ups among the study interventions included in this review, further research is required to understand which elements of telephone follow-ups lead to effective outcomes. It is possible that decreased hospitalization occurred for youth receiving the FBCI intervention because of the sustained family empowerment that allowed parents of suicidal youth to feel confident managing suicide-related concerns at home or using outpatient support.

With all included studies having been conducted in the United States, there is a need for research that investigates Emergency Department-based or initiated youth suicide prevention interventions in other countries and using a critical global health lens. Further, we recommend that future RCT studies give careful consideration to the primary outcome of the study. In this review, we have found that depression or depressive symptoms as well as family system functioning may be more modifiable outcomes, while suicide-related behaviours may be too distal from the study intervention. Lastly, we recommend robust youth engagement in study design and evaluation for future RCTs to ethically collaborate with and learn from the population who will receive the intervention.

## 6. Limitations

An important limitation of this review is that a meta-analysis could not be conducted due to the heterogeneity of the data. This is, in part, because of the selected inclusion and exclusion criteria which allowed for variance among study populations; youth presentations and chief complaints in the Emergency Department; and study interventions. Furthermore, this review only included studies with youth participants who were between 10 to 18 years of age. While the included population aligned with the scope of our systematic review, given recent evidence demonstrating that 43.1% of Emergency Department visits for suicidal ideation or following a suicide attempt in the United States were among children aged 5 to younger than 11 years of age [4], future reviews should aim to widen the age range to include this population. Although this updated review yielded only five articles, it is encouraging that 57 ongoing RCT studies are investigating youth suicide prevention interventions (S1 Table), representing an optimistic outlook for clinical practice and youths’ outcomes.

## 7. Conclusion

These results demonstrate that despite an urgent need for clinical guidelines that can address the rapidly increasing number of Emergency Department presentations for suicide-related concerns among youth, there is currently insufficient evidence of effectiveness of interventions upon which clinical guidelines can be made with confidence. As such, this area of clinical care remains in evolution. The RCT studies included in this review have trialed novel youth-targeted and family-targeted Emergency Department-based or initiated youth suicide prevention interventions with some demonstrating significantly improved outcomes for either decreasing depressive symptoms, hospitalization recidivism, and/or increasing family empowerment [26, 28]. None of the included studies, however, were effective in reducing suicidal ideation or suicide attempts with statistical significance. Further research is needed that is focused on improving suicidality, including related risk factors, as an important outcome among this population within the context of the Emergency Department.

## Supporting information

S1 Table Full text articles excluded with reasons (n = 141). (DOCX)

S1 Appendix MEDLINE search strategy output. (DOCX)

S1 Checklist (DOCX)

## References

[1] National Institute of Mental Health. 2020 [cited 2022 Aug 23]. Available from: https://www.nimh.nih.gov/health/statistics/suicide.shtml

[2] Statistics Canada. 2022 [cited 2022 Aug 23]. Available from: Table 13-10-0394-01 Leading causes of death, total population, by age group. 10.25318/1310039401-eng

[3] Bardach NS, Coker TR, Zima BT, Murphy JM, Knapp P, Richardson LP, et al. Common and costly hospitalizations for pediatric mental health disorders. Pediatrics (Evanston). 2014; 133(4), 602–609. doi: 10.1542/peds.2013-3165 24639270 PMC3966505

[4] Burstein B, Agostino H, & Greenfield B. Suicidal attempts and ideation among children and adolescents in US Emergency Departments, 2007–2015. JAMA Pediatrics. 2019; 173(6), 598–600. doi: 10.1001/jamapediatrics.2019.0464 30958529 PMC6547071

[5] Gilley M, Sivilotti MLA, Juurlink DN, Macdonald E, Yao Z, & Finkelstein Y. Trends of intentional drug overdose among youth: A population-based cohort study. Clinical Toxicology. 2019; 1–5. doi: 10.1080/15563650.2019.1687900 31760804

[6] Plemmons G, Hall M, Doupnik S, Gay J, Brown C, Browning W, et al. Hospitalization for suicide ideation or attempt: 2008–2015. Pediatrics (Evanston). 2018; 141(6), e20172426–. doi: 10.1542/peds.2017-2426 29769243

[7] Ting SA, Sullivan AF, Boudreaux ED, Miller I, & Camargo CA. Trends in US emergency department visits for attempted suicide and self-inflicted injury, 1993–2008. General Hospital Psychiatry. 2012; 34(5), 557–565. doi: 10.1016/j.genhosppsych.2012.03.020 22554432 PMC3428496

[8] Barzilay S, Apter A. Recent research advances in identification and prevention of youth suicide risk. Current opinion in psychiatry. 2022;35(6):395–400. doi: 10.1097/YCO.0000000000000816 35959553

[9] Hill RM, Rufino K, Kurian S, Saxena J, Saxena K, Williams L. Suicide Ideation and Attempts in a Pediatric Emergency Department Before and During COVID-19. Pediatrics (Evanston). 2021;147(3):1–. doi: 10.1542/peds.2020-029280 33328339

[10] Ridout KK, Alavi M, Ridout SJ, Koshy MT, Awsare S, Harris B, et al. Emergency Department Encounters Among Youth With Suicidal Thoughts or Behaviors During the COVID-19 Pandemic. JAMA psychiatry (Chicago, Ill). 2021;78(12):1319–28. doi: 10.1001/jamapsychiatry.2021.2457 34468724 PMC8411357

[11] Turner BJ, Robillard CL, Ames ME, Craig SG. Prevalence and Correlates of Suicidal Ideation and Deliberate Self-harm in Canadian Adolescents During the Coronavirus Disease 2019 Pandemic. Canadian journal of psychiatry. 2022;67(5):403–6. doi: 10.1177/07067437211036612 34378420 PMC9065494

[12] Bridge JA, Ruch DA, Sheftall AH, Hahm HC, O’Keefe VM, Fontanella CA, et al. Youth Suicide During the First Year of the COVID-19 Pandemic. Pediatrics (Evanston). 2023;151(3). doi: 10.1542/peds.2022-058375 36789551 PMC10227859

[13] Hawton K, & Harriss L. Deliberate self-harm in young people: characteristics and subsequent mortality in a 20-year cohort of patients presenting to hospital. The Journal of Clinical Psychiatry. 2007; 68(10), 1574–1583. doi: 10.4088/JCP.v68n1017 17960975

[14] Bennett K, Rhodes AE, Duda S, Cheung AH, Manassis K, Links P, et al. A youth suicide prevention plan for Canada: A aystematic review of reviews. The Canadian Journal of Psychiatry. 2015; 60(6), 245–257. 26175322 10.1177/070674371506000603 PMC4501582

[15] Asarnow J, Baraff L, Berk M, Grob C, Devich-Navarro M, Suddath R, et al. An Emergency Department intervention for linking pediatric suicidal patients to follow-up mental health treatment. Psychiatric Services. 2011; 62(11), 1303–1309. doi: 10.1176/ps.62.11.pss6211_1303 22211209 PMC3251923

[16] Brent D, Mcmakin D, Kennard B, Goldstein T, Mayes T, & Douaihy A. Protecting adolescents from self-harm: A critical review of intervention studies. Journal of the American Academy of Child & Adolescent Psychiatry. 2013; 52(12), 1260–1271. doi: 10.1016/j.jaac.2013.09.009 24290459 PMC3873716

[17] Hughes J, & Asarnow J. Family intervention strategies for adolescent depression. Pediatric Annals. 2011; 40(6), 314–318. doi: 10.3928/00904481-20110512-07 21678890

[18] Newton A, Hamm M, Bethell J, Rhodes A, Bryan C, Tjosvold L, et al. Pediatric suicide-related presentations: A systematic review of mental health care in the Emergency Department. Annals of Emergency Medicine. 2010; 56(6), 649–659.e2. doi: 10.1016/j.annemergmed.2010.02.026 20381916 PMC3012108

[19] Virk F, Waine J, Berry C. A rapid review of emergency department interventions for children and young people presenting with suicidal ideation. BJPsych open. 2022;8(2):e56–e56. doi: 10.1192/bjo.2022.21 35241211 PMC8935937

[20] Shamseer L, Moher D, Clarke M, Ghersi D, Liberati A, Petticrew M, et al. Preferred reporting items for systematic review and meta-analysis protocols (PRISMA-P) 2015: elaboration and explanation. BMJ. 2015; Jan 2;349(jan02 1):g7647. 10.1136/bmj.g7647 25555855

[21] Campbell M. Synthesis without meta-analysis (SWiM) in systematic reviews: reporting guideline. BMJ. 2020; 368, l6890–l6890. doi: 10.1136/bmj.l6890 31948937 PMC7190266

[22] Covidence systematic review software, Veritas Health Innovation, Melbourne, Australia. [cited 2022 Aug 23]. Available from: www.covidence.org

[23] McHugh C, Chun Lee R, Hermens D, Corderoy A, Large M, & Hickie I. Impulsivity in the self-harm and suicidal behavior of young people: A systematic review and meta-analysis. Journal of Psychiatric Research. 2019; 116, 51–60. doi: 10.1016/j.jpsychires.2019.05.012 31195164

[24] Cochrane Training. Chapter 8: Assessing risk of bias in a randomized trial. 2022 [cited 2022 Aug 23]. Available from: /handbook/current/chapter-08

[25] King C, Hill R, Wynne H, & Cunningham R. Adolescent suicide risk screening: The effect of communication about type of follow-up on adolescents’ screening responses. Journal of Clinical Child and Adolescent Psychology. 2012; 41(4), 508–515. doi: 10.1080/15374416.2012.680188 22540534 PMC3790145

[26] King C, Gipson P, Horwitz A, & Opperman K. Teen Options for Change: An intervention for young emergency patients who screen positive for suicide risk. Psychiatric Services. 2015; 66(1), 97–100. doi: 10.1176/appi.ps.201300347 25321886 PMC4346207

[27] Grupp-Phelan J, Stevens J, Boyd S, Cohen D, Ammerman R, Liddy-Hicks S, et al. Effect of a motivational interviewing-based intervention on initiation of mental health treatment and mental health after an Emergency Department visit among suicidal adolescents: A randomized clinical trial. JAMA Network Open. 2019; 2(12), e1917941–e1917941. doi: 10.1001/jamanetworkopen.2019.17941 31860104 PMC6991223

[28] Wharff E, Ginnis K, Ross A, White E, White M, & Forbes P. Family-Based Crisis Intervention with suicidal adolescents: A randomized clinical trial. Pediatric Emergency Care. 2019; 35(3), 170–175. doi: 10.1097/PEC.0000000000001076 28248838

[29] Anastasia T, Humphries‐Wadsworth T, Pepper C, & Pearson T. Family Centered Brief Intensive Treatment: A pilot study of an outpatient treatment for acute suicidal ideation. Suicide & Life-Threatening Behavior, vol. 45, no. 1, Guilford Publications, Inc, 2015; pp. 78–83, doi: 10.1111/sltb.12114 25169208

[30] Pineda D, & Dadds M. Family intervention for adolescents with suicidal behavior: A randomized controlled trial and mediation analysis. Journal of the American Academy of Child and Adolescent Psychiatry. 2013; 52(8), 851–862. doi: 10.1016/j.jaac.2013.05.015 23880495

[31] Wells K, & Heilbron N. Family-based cognitive-behavioral treatments for suicidal adolescents and their integration with individual treatment. Cognitive and Behavioral Practice, vol. 19, no. 2, Elsevier Ltd, 2012; pp. 301–14, doi: 10.1016/j.cbpra.2011.06.004

[32] Shields L, Pratt J, & Hunter J. Family centred care: a review of qualitative studies. Journal of Clinical Nursing. 2006; 15(10), 1317–1323. doi: 10.1111/j.1365-2702.2006.01433.x 16968436

[33] Holliday C, Vandermause R. Teen experiences following a suicide attempt. Archives of psychiatric nursing. 2015;29(3):168–73. doi: 10.1016/j.apnu.2015.02.001 26001716

