# Supplementary material for: Effectiveness of emergency department-based and initiated youth suicide prevention interventions: A systematic review
Source: PLoS One. 2023 Dec 5;18(12):e0289035. doi: 10.1371/journal.pone.0289035 (PMC10697510; doi:10.1371/journal.pone.0289035)
Supplement: S1 Table — (DOCX) [file pone.0289035.s001.docx]

**Full Text Articles Excluded with Reasons (n=141)**

| ***Wrong patient population*** | | | | | | |
| --- | --- | --- | --- | --- | --- | --- |
| **Authors** | **Year** | **Title** | **Journal** | **Volume** | **Issue** | **Pages** |
| Amadéo S, Rereao M, Mologne A, Favro P, Nguyen NL, Jehel L, Milner A, Kolves K, Leo DD. | 2015 | Testing brief intervention and phone contact among subjects with suicidal behavior: a randomized controlled trial in French Polynesia in the frames of the World Health Organization/Suicide Trends in At-Risk Territories study | Mental Illness | 7 | 2 |  |
| Andreoli A, Burnand Y, Cochennec MF, Ohlendorf P, Frambati L, Gaudry-Maire D, Clemente TD, Hourton G, Lorillard S, Canuto A, Frances A. | 2016 | Disappointed love and suicide: A randomized controlled trial of “abandonment psychotherapy” among borderline patients | Journal of Personality Disorders | 30 | 2 | 271-287 |
| Arvilommi P, Valkonen J, Ruishalme O, Kukkonen M, Sihvola H, Lindholm L, Suominen K, Gaily-Luoma S, Isometsa E. | 2022 | A randomized clinical trial of the ASSIP vs. crisis counselling in preventing suicide attempt repetition: a two-year follow-up study | Nordic Journal of Psychiatry |  |  |  |
| Barber C, Azrael D, Berrigan J, Betz ME, Brandspigel S, Runyan C, Salhi C, Vriniotis M, Miller M. | 2022 | Selection and Use of Firearm and Medication Locking Devices in a Lethal Means Counseling Intervention | Crisis: The Journal of Crisis Intervention and Suicide Prevention |  |  |  |
| Beautrais AL, Gibb SJ, Faulkner A, Fergusson DM, Mulder RT. | 2010 | Postcard intervention for repeat self-harm:  randomised controlled trial | The British Journal of Psychiatry | 197 | 1 | 55-60 |
| Bennewith O, Stocks N, Gunnell D, Peters TJ, Evans MO, Sharp DJ. | 2002 | General practice based intervention to prevent repeat episodes of deliberate self harm: cluster randomised controlled trial | BMJ | 324 |  |  |
| Bertolote JM, Fleischmann A, Leo DD, Phillips MR, Botega NJ, Vijayakumar L, Silva DD, Schlebusch L, Nguyen VT, Sisask M, Bolhari J, Wasserman D. | 2010 | Data from Emergency Care Settings in Five Culturally Different Low- and Middle-Income Countries Participating in the WHO SUPRE-MISS Study | Crisis | 31 | 4 | 194-201 |
| Brown GK, Have TT, Henriques GR, Xie SX, Hollander JE, Beck AT. | 2005 | Cognitive Therapy for the Prevention  of Suicide Attempts  A Randomized Controlled Trial | JAMA | 292 | 5 |  |
| Clarke T, Baker P, Watts CJ, Williams K, Feldman RA, Sherr L. | 2002 | Self-harm in adults: A randomised controlled trial of nurse-led case management versus routine care only | Journal of Mental Health | 11 | 2 | 167-176 |
| Crawford MJ, Csipke E, Brown A, Reid S, Nilsen K, Redhead J, Touquet R. | 2010 | The effect of referral for brief intervention for alcohol misuse on repetition of deliberate self-harm: an exploratory randomized controlled trial | Psychological Medicine | 40 |  | 1821-1828 |
| Currier GW, Fisher SG, Caine ED. | 2010 | Mobile Crisis Team Intervention to Enhance Linkage of Discharged Suicidal Emergency Department Patients to Outpatient Psychiatric Services: A Randomized Controlled Trial | Academic Emergency Medicine | 17 | 1 | 36-43 |
| Currier GW, Fisher SG, Caine ED. | 2010 | Mobile Crisis Team Intervention to Enhance Linkage of Discharged Suicidal Emergency Department Patients to Outpatient Psychiatric Services: A Randomized Controlled Trial | Academic Emergency Medicine | 17 | 1 | 36-43 |
| Denchev P, Pearson JL, Allen MH, Claassen CA, Currier GW, Zatzick DF, Schoenbaum M. | 2018 | Modeling the Cost-Effectiveness of Interventions to Reduce Suicide Risk Among Hospital Emergency Department Patients | Psychiatric Services | 69 | 1 |  |
| Dimeff LA, Jobes DA, Koerner K, Kako N, Jerome T, Kelley-Brimer A, Boudreaux ED, Beadnell B, Goering P, Witterholt S, Melin G, Samike V, Schak KM. | 2021 | Using a Tablet-Based App to Deliver Evidence-Based Practices for Suicidal Patients in the Emergency Department: Pilot Randomized Controlled Trial | JMIR Mental Health | 8 | 3 |  |
| Dunlap LJ, Orme S, Zarkin GA, Arias SA, Miller IW, Camargo CA, Sullivan AF, Allen MH, Goldstein AB, Manton AP, Clark R, Boudreaux ED. | 2019 | Screening and Intervention for Suicide Prevention: A Cost-Effectiveness Analysis of the ED-SAFE Interventions | Psychiatry Services | 70 | 12 | 1082-1087 |
| Exbrayat S, Coudrot C, Gourdon X, Gay A, Sevos J, Pellet J, Trombert-Paviot B, Massoubre C. | 2017 | Effect of telephone follow-up on repeated suicide attempt in patients discharged from an emergency psychiatry department: a controlled study | BMC Psychiatry | 17 | 1 |  |
| Foster CE, Horwitz A, Thomas A, Opperman K, Gipson P, Burnside A, Stone DM, King CA. | 2017 | Connectedness to family, school, peers, and community in socially vulnerable adolescents | Child and Youth Services Review | 81 |  | 321-331 |
| Furuno T, Nakagawa M, Hino K, Yamada T, Kawashima Y, Matsuoka Y, Shirakawa O, Ishizika N, Yonemoto N, Kawanishi C, Hirayasu Y. | 2018 | Effectiveness of assertive case management on repeat self-harm in patients admitted for suicide attempt: Findings from ACTION-J study | Journal of Affective Disorders | 225 |  | 460-465 |
| Guthrie E, Kapur N, Mackway-Jones K, Chew-Graham C, Moorey J, Mendel E, Marino-Francis F, Sanderson S, Turpin C, Boddy G, Tomenson B. | 2001 | Randomised controlled trial of brief psychological intervention after deliberate self poisoning | BMJ | 323 |  |  |
| Hatcher S, Coupe N, Wikiriwhi K, Durie SM, Pillai A. | 2016 | Te Ira Tangata: a Zelen randomised controlled trial of a culturally informed treatment compared to treatment as usual in Ma ̄ori who present to hospital after self-harm | Soc Psychiatry Psychiatr Epidemiol | 51 |  | 885-894 |
| Inui-Yukawa M, Miyaoka H, Yamamoto K, Kamijo Y, Takai M, Yonemoto N, Kawanishi C, Otsuka K, Tachikawa H, Hirayasu Y. | 2021 | Effectiveness of assertive case management for patients with suicidal intent | Psychiatry Research | 304 |  |  |
| Johnson S, Lamb D, Marston L, Osborn D, Mason O, Henderson C, Ambler G, Milton A, Davidson M, Christoforou M, Sullivan S, Hunter R, Hindle D, Paterson B, Leverton M, Piotrowski J, Forsyth R, Mosse L, Goater N, Kelly K, Lean M, Piling S, Morant N, Lloyd-Evans B. | 2018 | Peer-supported self-management for people discharged from a mental health crisis team: a randomised controlled trial | The Lancet | 392 |  | 409-418 |
| Kim MH, Lee J, Noh H, Hong JP, Kim H, Cha YS, Ahn JS, Chang SJ, Min S. | 2020 | Effectiveness of a Flexible and Continuous Case Management Program for Suicide Attempters | International Journal of Environmental Research and Public Health | 17 | 7 | 2599 |
| Lin YC, Liu SI, Chen SC, Sun FJ, Huang HC, Huang CR, Chiu YC. | 2020 | Brief Cognitive-based Psychosocial Intervention and Case Management for Suicide Attempters Discharged from the Emergency Department in Taipei, Taiwan: A Randomized Controlled Study | Suicide & life-threatening behavior |  |  |  |
| Linehan MM, Comtois KA, Murray AM, Brown MZ, Gallop RJ, Heard HL, Korslund KR, Tutek DA, Reynolds SK, Lindenboim N. | 2006 | Two-Year Randomized Controlled Trial and Follow-up of Dialectical Behavior Therapy vs Therapy by Experts for Suicidal Behaviors and Borderline Personality Disorder | Arch Gen Psychiatry | 63 |  | 757-766 |
| Linehan MM, Korslund KE, Harned MS, Gallop RJ, Lungu A, Neacsiu AD, McDavid J, Comtois KA, Murray-Gregory AM. | 2015 | Dialectical Behavior Therapy for High Suicide Risk  in Individuals With Borderline Personality Disorder  A Randomized Clinical Trial and Component Analysis | JAMA Psychiatry | 72 | 5 |  |
| Mouaffak F, Marchand A, Castaigne E, Arnoux A, Hardy P. | 2015 | OSTA program: A French follow up intervention program for suicide prevention | Psychiatry research | 230 | 9 | 913-8 |
| Owens D, Wright-Hughes A, Graham L, Blenkiro P, Burton K, Collinson M, Farrin A, Hatcher S, Martin K, O’Dwyer J, Pembroke L, Protheroe D, Tubeuf S, House A. | 2020 | Problem-solving therapy rather than treatment as usual for adults after self-harm: a pragmatic, feasibility, randomised controlled trial (the MIDSHIPS trial) | Pilot and Feasibility Studies | 6 | 119 |  |
| Runyan CW, Brandspigel S, Barber CW, Betz M, Azrael D, Miller M. | 2019 | Lessons learned in conducting youth suicide prevention research in emergency departments | Injury Prevention | 26 |  | 159-163 |
| Vaiva G, Durocq F, Meyer P, Mathieu D, Philippe A, Libersa C, Goudemand M. | 2006 | Effect of telephone contact on further suicide attempts in patients discharged from an emergency department: randomised controlled study | BMJ | 332 |  | 1241-5 |
| Wei S, Liu L, Bi B, Li H, Hou J, Tan S, Chen X, Chen W, Jia X, Dong G, Qin, Liu Y. | 2013 | An intervention and follow-up study following a suicide attempt in the emergency departments of four general hospitals in Shenyang, China | Crisis | 34 | 2 | 107-15 |
| Welu TC | 1977 | A follow-up program for suicide attempters: evaluation of effectiveness | Suicide and Life-Threatening Behavior | 7 | 1 | 17-20 |
| Westling S, Daukantaitė D, Liljedahl S, Oh Y, Westrin A, Flyckt L, Helleman M. | 2019 | Effect of Brief Admission to Hospital by Self-referral for Individuals Who Self-harm and Are at Risk of Suicide | JAMA Network Open | 2 | 6 |  |
| ***Registered Trial*** | | | | | | |
| **Authors** | **Year** | **Title** | **Journal** | **Volume** | **Issue** | **Pages** |
| Bilokas VV, Hains AR, Allan JA, Lago L, Sng R. | 2019 | Community-based aftercare following an emergency department presentation for attempted suicide or high risk for suicide: study protocol for a non-randomised controlled trial | BMC Public Health | 19 | 1 |  |
| Boudreaux ED, Stanley B, Green KL, Galfalvy H, Brown GK. | 2021 | A randomized, controlled trial of the safety planning intervention: Research design and methods | Contemporary Clinical Trials | 103 |  |  |
| Deluca P, Coulton S, Fasihul Alam M, Boniface S, Donoghue K, Gilvarry E, Kaner E, Lynch E, Maconochie I, McArdle P, McGovern R, Newbury-Birch D, Patton R, Pellatt-Higgins T, Phillips C, Phillips T, Pockett R, Russell IT, Strang J, Drummond C. | 2020 | Screening and brief interventions for adolescent alcohol use disorders presenting through emergency departments: a research programme including two RCTs | National Institute for Health Research Journals Library | 8 | 2 |  |
| Freedman S, Thull-Freedman J, Lightbody T, Prisnie K, Wright B, Coulombe A, Anderson LM, Stang AS, Mikrogianakis A, VanRiper L, Stubbs M, Newton A, PERC. | 2020 | Introducing an innovative model of acute paediatric mental health and addictions care to paediatric emergency departments: a protocol for a multicentre prospective cohort study | BMJ Open Quality | 9 | 4 |  |
| Hatcher S, Heisel M, Ayonrinde O, Campbell JK, Colman I, Corsi DJ, Edgar NE, Gillett L, Kennedy SH, Hunt SL, Links P, MacLean S, Mehta V, Mushquash C, Raimundo A, Rizvi SJ, Saskin R, Schaffer A, Sidahmed A, Sinyor M, Soares C, Taljaard M, Testa V, Vaillancourt C. | 2020 | The BEACON study: protocol for a cohort study as part of an evaluation of the effectiveness of smartphone-assisted problem-solving therapy in men who present with intentional self-harm to emergency departments in Ontario | Trials | 21 | 1 | 925 |
| Husain N, Tofique S, Chaudhry IB, et al. | 2022 | Youth Culturally adapted Manual Assisted Problem Solving Training (YCMAP) in Pakistani adolescent with a history of self-harm: protocol for multicentre clinical and cost-effectiveness randomised controlled trial | BMJ Open | 12 | 5 |  |
| Jolley S, Browning S, Corrigall R, Laurens KR, Hirsch C, Bracegirdle K, Gin K, Muccio F, Stewart C, Banerjea P, Kuipers E, Garety P, Byrne M, Onwumere J, Achilla E, McCrone P, Emsley R. | 2017 | Coping with Unusual ExperienceS for 12-18 year olds (CUES+): a transdiagnostic randomised controlled trial of the effectiveness of cognitive therapy in reducing distress associated with unusual experiences in adolescent mental health services: study protocol for a randomised controlled trial | Trials | 18 |  | 1-14 |
| Kawanishi, C. | 2021 | The strategic research for care for suicide attempters and its social implementation in Japan | Asia-Pacific Psychiatry | 13 |  |  |
| Korczak DJ, Finkelstein Y, Barwick M, Chaim G, Cleverley K, Henderson J, Monga S, Moretti ME, Willan A, Szatmari P. | 2020 | A suicide prevention strategy for youth presenting to the emergency department with suicide related behaviour: protocol for a randomized controlled trial | BMC Psychiatry | 20 | 1 |  |
| Registration number: ACTRN12617000043336 | 2017 | Mass media campaign material designed to prevent youth suicide: a randomised controlled trial |  |  |  |  |
| Registration number: ACTRN12621001123831 | 2021 | Bipolar early interventions using new digital technologies (BLEND): a feasibility trial |  |  |  |  |
| Registration number: ACTRN12620000893909 | 2020 | Intensive Trauma-Focused Cognitive Behavioural Therapy for the treatment of Post-Traumatic Stress Disorder (PTSD) in an acute adolescent mental health unit and 3-month follow-up |  |  |  |  |
| Registration number: ACTRN12621001221842 | 2021 | Mobile phone access to Psychological Therapies (IAPT-M) for people presenting to the Emergency Department with suicidal behaviour |  |  |  |  |
| Registration number: ACTRN12617000457347 | 2017 | LifeSpan: a whole-population stepped-wedge cluster-randomized trial of a multilevel systems approach to reduce suicide attempts and deaths in four NSW regions |  |  |  |  |
| Registration number: IRCT2013050813273N1 | 2014 | The effect of preventive interventions on suicide attempt |  |  |  |  |
| Registration number: IRCT20201026049155N1 | 2021 | Evaluation of the effect of repeated transcranial magnetic stimulation (RTMS) in reducing the percentage of suicidal ideation in depressed patients compared with the control group |  |  |  |  |
| Registration number: ISRCTN14748840 | 2021 | An assessment of the effectiveness of Psychodynamic Interpersonal Therapy in reducing the repetition of self-harm in adults presenting to an emergency department with acute self-harm (history of 3 or fewer episodes in the last 12 month) |  |  |  |  |
| Registration number: NCT00183157 | 2005 | Project RAP: reaching Adolescents for Prevention |  |  |  |  |
| Registration number: NCT00218725 | 2005 | Effectiveness of Cognitive Therapy for Suicide Attempters With Drug Dependence Disorder |  |  |  |  |
| Registration number: NCT00641498 | 2008 | Effectiveness of Standard Emergency Department Psychiatric Treatment Associated With Treatment Delivery by a Suicide Prevention Center |  |  |  |  |
| Registration number: NCT01779414 | 2013 | STAT-ED: suicidal Teens Accessing Treatment After an Emergency Department Visit |  |  |  |  |
| Registration number: NCT01823120 | 2013 | Text Message Intervention to Reduce Repeat Self-harm |  |  |  |  |
| Registration number: NCT02510508 | 2015 | Group Version of CRAFT Compared to Self-Directed CRAFT Delivery and Non-intervention: a Three-armed RCT |  |  |  |  |
| Registration number: NCT02718248 | 2015 | Ottawa Suicide Prevention in Men Pilot Study |  |  |  |  |
| Registration number: NCT02751203 | 2016 | Make Safe Happen App Evaluation Study |  |  |  |  |
| Registration number: NCT02759172 | 2016 | Suicide Prevention Intervention for At-Risk Individuals in Transition |  |  |  |  |
| Registration number: NCT02877316 | 2016 | MYPLAN - Effectiveness of a Safety Plan App to Manage Crisis of Persons at Risk of Suicide |  |  |  |  |
| Registration number: NCT03092271 | 2017 | Randomized Trial of Stepped Care for Suicide Prevention in Teens and Young Adults |  |  |  |  |
| Registration number: NCT03143283 | 2017 | An ED-based RCT of Lethal Means Counseling for Parents of At-Risk Youth |  |  |  |  |
| Registration number: NCT03514004 | 2018 | Using Information and Communication Technologies to Prevent Suicide in Chile |  |  |  |  |
| Registration number: NCT03653637 | 2018 | Group ("Project Life Force") vs. Individual Suicide Safety Planning RCT |  |  |  |  |
| Registration number: NCT03894462 | 2019 | Effectiveness of a Targeted Brief Intervention for Recent Suicide Attempt Survivors |  |  |  |  |
| Registration number: NCT03924037 | 2019 | Zero Suicide Plus KICKS |  |  |  |  |
| Registration number: NCT03940716 | 2019 | Project IntERact Study |  |  |  |  |
| Registration number: NCT03975881 | 2019 | Study for the Use Smartphone Application to Prevent Suicidal Relapse Among 15-35 Years-old With Previous Suicide Attempted |  |  |  |  |
| Registration number: NCT04089254 | 2019 | Suicide Treatment Alternatives for Teens |  |  |  |  |
| Registration number: NCT04658420 | 2020 | A Single Ketamine Infusion Combined with Music for Suicidal Ideation |  |  |  |  |
| Registration number: NCT04642157 | 2020 | Online Referral and Intervention to Prevent Adolescent and Young Adult Suicide |  |  |  |  |
| Registration number: NCT04260607 | 2020 | Initiating Ketamine in Acutely Suicidal Patients in the Emergency Department |  |  |  |  |
| Registration number: NCT04939727 | 2021 | Suicide Prediction and Prevention for People at Risk for Opioid Use Disorder: Supplement to COMPUTE 2.0 |  |  |  |  |
| Registration number: NCT04669665 | 2020 | A Study of SLS-002 (Intranasal Racemic Ketamine) in Adults with Major Depressive Disorder at Imminent risk of Suicide |  |  |  |  |
| Registration number: NCT05334381 | 2022 | Navigating Mental Health Treatment for Black Youth |  |  |  |  |
| Registration number: NCT04498143 | 2020 | Single-Session Intervention Targeting Self-Injurious Behavior in Adolescents |  |  |  |  |
| Registration number: NCT04775160 | 2021 | Smartphone-based Ecological Momentary Intervention for Suicide Prevention: a Randomised Clinical Trial |  |  |  |  |
| Registration number: NCT05307432 | 2022 | Safety Planning Intervention Telehealth Service Model in Emergency Departments |  |  |  |  |
| Registration number: NCT05304065 | 2022 | Youth Partners in Care for Suicide Prevention |  |  |  |  |
| Registration number: NCT04893447 | 2021 | Suicide Prevention Among Recipients of Care |  |  |  |  |
| Registration number: NCT05217706 | 2022 | Low-Dose Intravenous Ketamine for Adolescents With Depression and Suicidal Ideation in the Emergency Department |  |  |  |  |
| Registration number: NCT04955470 |  | Ketamine Infusion for Rapid Reduction of Suicidality in Pediatrics |  |  |  |  |
| Registration number: NCT04414774 | 2020 | Assessing the Effectiveness of a CBT-based App in Reducing Suicidal Ideation |  |  |  |  |
| Registration number: NCT04640636 | 2020 | IM Ketamine vs Midazolam for Suicidal ER Patients |  |  |  |  |
| Rosebrock H, Chen N, Tye M, Mackinnon A, Calear AL, Batterham PJ, Maple M, Rasmussen VM, Schroeder L, Cutler H, Shand F. | 2020 | Study protocol for a mixed methods prospective cohort study to explore experiences of care following a suicidal crisis in the Australian healthcare system | BMJ Open | 10 | 8 |  |
| Stapelberg NJC, Bowman C, Woerwag-Mehta S, Walker S, Davies A, Hughes I, et al. | 2021 | A lived experience co-designed study protocol for a randomised control trial: the Attempted Suicide Short Intervention Program (ASSIP) or Brief Cognitive Behavioural Therapy as additional interventions after a suicide attempt compared to a standard Suicide Prevention Pathway (SPP) |  |  |  |  |
| Stanley B, Labouliere CD, Brown GK, Green KL, Galfalvy HC, Finnerty MT, Vasan P, Cummings AK, Wainberg M, Carruthers JW, Dixon LB. | 2021 | Zero suicide implementation-effectiveness trial study protocol in outpatient behavioral health using the A-I-M suicide prevention model | Contemporary Clinical Trials | 100 |  |  |
| Stapelberg NJC, Bowman C, Woerwag-Mehta S, Walker S, Davies A, Hughes I, Michel K, Pisani AR, Engelen HV, Delos M, Hageman T, Fullerton-Smith K, Krishnaiah R, McDowell S, Cameron A, Scales TL, Dillon C, Gigante T, Heddle C, Mudge N, Zappa A, Edwards M, Gutjahr S, Joshi H, Turner K. | 2021 | A lived experience co-designed study protocol for a randomised control trial: the Attempted Suicide Short Intervention Program (ASSIP) or Brief Cognitive Behavioural Therapy as additional interventions after a suicide attempt compared to a standard Suicide Prevention Pathway (SPP) | Trials | 22 | 1 |  |
| Stevens GJ, Hammond TE, Brownhill S, Anand M, Riva A, Hawkins J, Chapman T, Baldacchino, R, Micallef, JA, Adepalli, J. | 2019 | SMS SOS: a randomized controlled trial to reduce self-harm and suicide attempts using SMS text messaging |  |  |  |  |
| Van Oenen FJ, Schipper S, Van R, Schoevers R, Visch I, Peen J, Dekker J. | 2013 | Efficacy of immediate patient feedback in emergency psychiatry: a randomized controlled trial in a crisis intervention & brief therapy team |  |  |  |  |
| ***Wrong indication*** | | | | | | |
| **Authors** | **Year** | **Title** | **Journal** | **Volume** | **Issue** | **Pages** |
| Arterberry BJ, Davis AK, Walton MA, Bonar EE, Cunningham RM, Blow FC. | 2019 | Predictors of empirically derived substance use patterns among sexual minority groups presenting at an emergency department | Addictive Behaviors | 96 |  | 76-81 |
| Busby DR, King CA, Brent D, Grupp-Phelan J, Gould M, Page K, Casper TC, PECARN. | 2020 | Adolescents’ Engagement with Crisis Hotline Risk-management Services: A Report from the Emergency Department Screen for Teen Suicide Risk (ED-STARS) Study | Suicide and Life-Threatening Behavior | 50 | 1 |  |
| Giles LL. | 2019 | 4.2 Utilizing a trauma-informed therapeutic intervention in the Emergency Department for risk assessment | Journal of the American Academy of Child & Adolescent Psychiatry | 58 | 105 |  |
| Gruat G, Cottencin O, Ducrocq F, Duhem S, Vaiva G. | 2010 | Vécu subjectif du recontact téléphonique après  tentative de suicide | L’Encéphale | 36 |  |  |
| Grupp-Phelan J, McGuire L, Husky MM, Olfson M. | 2012 | A Randomized Controlled Trial to Engage in Care of Adolescent Emergency Department Patients With Mental Health Problems That Increase Suicide Risk | Pediatric Emergency Care | 28 | 12 |  |
| Hughes JL, Asarnow JR. | 2013 | Enhanced Mental Health Interventions in the Emergency Department: Suicide and Suicide Attempt Prevention | Clinical Pediatric Emergency Medicine | 14 | 1 | 28-34 |
| Latimer EA, Garièoy G, Greenfield B. | 2014 | Cost-effectiveness of a rapid response team intervention for suicidal youth presenting at an emergency department | La Revue Canadienne de psychatrie | 59 | 6 |  |
| Tracey M, Finkelstein Y, Schachter R, Cleverley K, Monga S, Barwick M, Szatmari P, Moretti ME, Willan A, Henderson J, Korczak D. | 2020 | Recruitment of adolescents with suicidal ideation in the emergency department: lessons from a randomized controlled pilot trial of a youth suicide prevention intervention | BMC Medical Research Methodology | 20 | 1 | 231 |
| Walsh K, Badour CL, Zuromski KL, Gilmore AK, Kilpatrick DG, Acierno R, Resnick HS. | 2021 | A Secondary Analysis of a Brief Video Intervention of Suicidal Ideation Among Recent Rape Victims | Psychological Services | 18 | 4 | 703-708 |
| ***Wrong study design*** | | | | | | |
| **Authors** | **Year** | **Title** | **Journal** | **Volume** | **Issue** | **Pages** |
| Alonzo D, Stanley, B. | 2013 | A novel intervention for treatment of suicidal individuals | Psychiatric services | 64 | 5 |  |
| Apple RW, Patel K, Smith Z. | 2020 | The role of integrated behavioural health (IBH) in suicide prevention | International Journal of Social Psychiatry | 63 | 4 | 359-375 |
| AriasdelaTorre J, Ronaldson A, Vilagut G, Serrano-Blanco A, Molina AJ, Martin V, Valderas JM, Dutta R, Dregan A, Alonso J. | 2021 | Improving suicide surveillance systems through the use of the Patient Health Questionnaire-9 | Journal of affective disorders | 293 |  | 71-72 |
| Asarnow JR, Berk MS. | 2009 | Family Intervention for Suicide Prevention: A Specialized Emergency Department Intervention for Suicidal Youths | Professional Psychology: Research and Practice | 40 | 2 | 118-125 |
| Asarnow JR, Goldston DB, Tunno AM, Inscoe AB, Pynoos R. | 2020 | Suicide, Self-Harm, & Traumatic Stress Exposure: A Trauma-Informed Approach to the Evaluation and Management of Suicide Risk | Evidence-Based Practice in Child and Adolescent Mental Health | 5 | 4 | 483-500 |
| Boudreaux ED, Larkin C, Ma Y, Li Y, Brown G, Stanley B, Pelletier L, Johnson SA. | 2020 | ED-safe 2: Improving suicide prevention through continuous quality improvement | Academic Emergency Medicine | 27 |  |  |
| Busby DR, King CA, Brent D, Grupp-Phelan J, Gould M, Page K, Casper TC, PECARN. | 2020 | Adolescents’ Engagement with Crisis Hotline Risk-management Services: A Report from the Emergency Department Screen for Teen Suicide Risk (ED-STARS) Study | Suicide and Life-Threatening Behavior | 50 | 1 |  |
| Cross M, Clarke T. | 2022 | Rapid response pathway united to reduce self-harm (RUSH): A case study of a pilot pathway for children and young people | Journal of Public Mental Health | 21 | 1 | 15-22 |
| Deykin EY, Hsieh CC, Joshi N, Mcnamarra JJ. | 1986 | Adolescent Suicidal and Self-Destructive Behavior | Journal of Adolescent Health Care | 7 |  | 88-95 |
| Dimeff LA, Jobes DA, Chalker SA, Piehl BM, Duvivier LL, Lok BC, Zalake MS, Chung J, Koerner K. | 2020 | A novel engagement of suicidality in the emergency department: Virtual Collaborative Assessment and Management of Suicidality | General Hospital Psychiatry | 63 |  | 119-126 |
| Esposito JM, Fein JA, Marshall J, Mitchell C, Aredas B, Zorc JJ, Rutman LE. | 2020 | Improving Mental Health Communication from the Pediatric Emergency Department to Primary Care | Pediatric Emergency Care | 36 | 9 | 424-429 |
| Fernández-Artamendi S, Al-Halabí S, Burón P, Rodríguez-Revuelta J, Garrido M, González-Blanco L, García-Álvarez L, García-Portilla P, Sáiz P, Bobes J. | 2019 | Prevention of recurrent suicidal behaviour: Case management and psychoeducation | Psicothema | 31 | 2 | 107-113 |
| Fernandez-Sevillano J, Errementeria L, Zorrilla I, Lopez P, Lopez S, Gonzalez-Pinto A. | 2020 | P.678 Suicide prevention by a telemedicine-based intervention: preeliminary results | European Neuropsychopharmacology | 40 |  |  |
| Gilmore AK, Hahn CK, Jaffe AE, Walsh K, Moreland AD, Ward-Ciesielski EF. | 2018 | Suicidal ideation among adults with a recent sexual assault: Prescription opioid use and prior sexual assault | Addictive Behaviors | 85 |  | 120-124 |
| Havens J, Marr MC. | 2015 | Models of emergency psychiatric care for children and adolescents: Moving from triage to meaningful engagement in mental health treatment | Soc Work | 57 | 2 | 133-143 |
| Legambi TF, Doede M, Michael K, Zaleski M. | 2021 | A Quality Improvement Project on Agitation Management in the Emergency Department | Journal of Emergency Nursing | 47 | 3 | 390-399 |
| López-Goñi JJ, Goñi-Sarriés A. | 2021 | Effectiveness of a telephone prevention programme on the recurrence of suicidal behaviour. One-year follow-up | Psychiatry Research | 302 |  |  |
| Martínez-Alés G, Jiménez-Sola E, Román-Mazuecos E, Sánchez-Castro MP, Dios CD, Rodríguez-Vega B, Bravo-Ortiz MF. | 2019 | An Emergency Department-Initiated Intervention to Lower Relapse Risk after Attempted Suicide | Suicide and Life-Threatening Behavior | 49 | 6 |  |
| Pestian JP, Grupp-Phelan J, Cohen KB, Meyers B, Richey LA, Matykiewiz P, Sorter MT. | 2016 | A Controlled Trial Using Natural Language Processing to Examine the Language of Suicidal Adolescents in the Emergency Department | Suicide and Life-Threatening Behavior | 46 | 2 |  |
| Ryan TC, Chambers S, Gravey M, Jay SY, Wilcox HC, Cwik M. | 2022 | A Brief Text-Messaging Intervention for Suicidal Youths After Emergency Department Discharge | PS in Advance |  |  |  |
| Sale E, Sandhu AS, VonDras S. | 2021 | Effectiveness of a Continuity-of-Care Model to Reduce Youth Suicidality: Preliminary Evidence From Kansas City, USA | Crisis: The Journal of Crisis Intervention and Suicide Prevention |  |  |  |
| Sedghy Z, Yoosefi N, Navidian A. | 2020 | The effect of motivational interviewing-based training on the rate of using mental health services and intensity of suicidal ideation in individuals with suicide attempt admitted to the emergency department | Journal of Education and Health Promotion | 9 |  |  |
| Taylor PJ, Fien K, Mulholland H, Duarte R, Dickson JM, Kullu C. | 2021 | Pilot service evaluation of a brief psychological therapy for self-harm in an emergency department: Hospital Outpatient Psychotherapy Engagement Service | Psychology and Psychotherapy: Theory, Research and Practice | 94 |  | 64-78 |
| Xu D, Zhang XL, Li XY, Niu YJ, Zhang YP, Wang SL, Yang FD, Cao KJ, Xu YC. | 2012 | Effectiveness of 18-month psychosocial intervention for suicide attempters | Chinese Mental Health Journal |  |  |  |
| ***Wrong setting*** | | | | | | |
| **Authors** | **Year** | **Title** | **Journal** | **Volume** | **Issue** | **Pages** |
| Diamond G, Wintersteen MB, Brown GK, Diamond G, Gallop R, Shelef K, Levy S. | 2010 | Attachment-Based Family Therapy for  Adolescents with Suicidal Ideation:  A Randomized Controlled Trial | Journal of the American Academy of Child and Adolescent Psychiatry | 49 | 2 |  |
| Goldston DB, Curry JF, Wells KC, Kaminer Y, Daniel SS, Esposito-Smythers C, Doyle O, Sapyta J, Tunno AM, Heilbron NC, Roley-Roberts M. | 2021 | Feasibility of an Integrated Treatment Approach for Youth with Depression, Suicide Attempts, and Substance Use Problems | Evidence-Based Practice in Child and Adolescent Mental Health | 6 | 2 | 155-172 |
| Huey SJ, Henggeler SW, Rowland MD, Halliday-Boykins CA, Cunningham PB, Pickrel SG, Edwards J. | 2004 | Multisystemic therapy effects on attempted suicide by youths presenting psychiatric emergencies | Journal of the American Academy of Child and Adolescent Psychiatry | 43 | 2 |  |
| Mehlum L, Tormoen AJ, Ramberg M, Haga E, Diep LM, Laberg S, Larsson BS, Stanley BH, Miller AL, Sund AM, Groholt B. | 2014 | Dialectical Behavior Therapy for Adolescents  With Repeated Suicidal and Self-harming  Behavior: A Randomized Trial | Journal of the American Academy of Child and Adolescent Psychiatry | 53 | 10 |  |
| Ougrin D, Corrigall R, Poale J, Zundel T, Sarhane M, Slater V, Stahl D, Reavey P, Byford S, Heslin M, Ivens J, Crommelin M, Abdulla Z, Hayes D, Middleton K, Nnadi B, Taylor E. | 2018 | Comparison of effectiveness and cost-effectiveness of an intensive community supported discharge service versus treatment as usual for adolescents with psychiatric emergencies: a randomised controlled trial | The Lancet | 5 |  |  |
| Spirito A, Boergers J, Donaldson D, Bishop D, Lewander W. | 2002 | An Intervention Trial to Improve Adherence to Community Treatment by Adolescents After a Suicide Attempt | Journal of the American Academy of Child and Adolescent Psychiatry | 41 | 4 |  |
| ***Wrong intervention*** | | | | | | |
| **Authors** | **Year** | **Title** | **Journal** | **Volume** | **Issue** | **Pages** |
| Cappelli M, Zemek R, Polihronis C, Thibedeau NR, Kennedy A, Gray C, Jabbour M, Reid S, Coutier P. | 2020 | The HEADS-ED  Evaluating the Clinical Use of a Brief, Action-Oriented, Pediatric Mental Health Screening Tool | Pediatric Emergency Care | 36 | 1 |  |
| Coshal S, Saunders J, Matorin AA, Shah AA. | 2017 | Evaluation of Depression and Suicidal Patients in the  Emergency Room | Psychiatric Clinics | 40 | 3 | 363-377 |
| ***Conference abstract*** | | | | | | |
| **Authors** | **Year** | **Title** | **Journal** | **Volume** | **Issue** | **Pages** |
| Barzman D, Combs J, Lin D. | 2021 | 44.4 What is the impact of suicide thought severity and other demographic factors on deciding treatment disposition in adolescent psychiatry emergency room visits? | Journal of the American Academy of Child and Adolescent Psychiatry |  |  |  |
| González-Pinto A, Fernández-Sevillano J, Gabilando A, Aristegui E, Roca R, Iruín Á, Martín JA, Martínez-Cengotitabengoa M, López P, Zorrilla I, López S. | 2019 | Telemedicine-based and integrated-care suicide prevention | 19^th^ International Conference on Integrated Care |  |  |  |
| Kim AK, Vakkalanka J, Tate J, Himadi E, Lee S. | 2020 | 405 Crisis Stabilization Unit Reduces Admission Rates for Suicidal Patients in a Midwest Emergency Department | Annals of Emergency Medicine | 76 |  |  |
| Tracey M, Rowney G, Pignatiello A, Monga S, Korczak D. | 2018 | Feasibility of a manualized family-based suicide prevention strategy to reduce adolescent suicide risk among emergency department referred youth: A quality improvement project | Paediatrics and Child Health (Canada) |  |  |  |
| ***Duplicate*** | | | | | | |
| Grupp-Phelan J, Stevens J, Boyd S, Cohen DM, Ammerman RT, Liddy-Hicks S, Heck K, Marcus SC, Stone L, Campo JV, Bridge JA. | 2019 | Effect of a motivational interviewing-based intervention on initiation of mental health treatment after an Emergency Department visit among suicidal adolescents: A randomized clinical trial | JAMA Network Open | 2 | 12 |  |
| Sale E, Sandhu AS, VonDras S. | 2021 | Effectiveness of a Continuity-of-Care Model to Reduce Youth Suicidality: Preliminary Evidence From Kansas City, USA | Crisis: The Journal of Crisis Intervention and Suicide Prevention |  |  |  |
| Wharff EA, Ginnis KB, Ross AM, White EM, White MT, Forbes PW. | 2019 | Family-Based Crisis Intervention With Suicidal Adolescents: a Randomized Clinical Trial | Pediatric Emergency Care | 35 | 3 |  |
| Wharff EA, Ginnis KB, Ross AM, White EM, White MT, Forbes PW. | 2019 | Family-Based Crisis Intervention With Suicidal Adolescents: a Randomized Clinical Trial | Pediatric Emergency Care | 35 | 3 |  |
