# Supplementary material for: Effectiveness of emergency department-based and initiated youth suicide prevention interventions: A systematic review
Source: PLoS One. 2023 Dec 5;18(12):e0289035. doi: 10.1371/journal.pone.0289035 (PMC10697510; doi:10.1371/journal.pone.0289035)
Supplement: S1 Appendix — (DOCX) [file pone.0289035.s002.docx]

**Appendix A**

MEDLINE Search Strategy Output

| 1 randomized controlled trial.pt.  2 controlled clinical trial.pt.  3 randomized.ab.  4 placebo.ab.  5 drug therapy.fs.  6 randomly.ab.  7 trial.ab.  8 groups.ab.  9 1 or 2 or 3 or 4 or 5 or 6 or 7 or 8  10 exp animals/ not humans.sh.  11 9 not 10  12 exp self-injurious behavior/  13 suicid*.tw,kf.  14 (attempt* adj suicid*).tw,kf.  15 ((Substance* or drug*) adj3 (us* or abuse* or problem* or consum* or overdose* or addict*)).tw,kf.  16 (((tak* own or plan* to end* own) adj3 life) or (deliberat* kill* adj3 self) or (self adj3 murder)).tw,kf.  17 (self adj3 harm).tw,kf.  18 (auto mutil* or (self adj3 injur* behav*) or (non suicid* self adj3 injur*) or (self adj3 inflict* violence)).tw,kf.  19 (emergency room* or emergency department* or emergency service*).tw,kf.  20 secondary prevention/ or preventive health services/  21 (prevent* or interrupt* or deter* or counter*).tw,kf.  22 screen*.tw,kf.  23 12 or 13 or 14 or 15 or 16 or 17 or 18  24 20 or 21 or 22  25 11 and 19 and 23 and 24 |
| --- |
